# Time-restricted feeding alters lipid and amino acid metabolite rhythmicity without perturbing clock gene expression

**DOI:** 10.1038/s41467-020-18412-w

**Published:** 2020-09-16

**Authors:** Leonidas S. Lundell, Evelyn B. Parr, Brooke L. Devlin, Lars R. Ingerslev, Ali Altıntaş, Shogo Sato, Paolo Sassone-Corsi, Romain Barrès, Juleen R. Zierath, John A. Hawley

**Affiliations:** 1 Novo Nordisk Foundation Center for Basic Metabolic Research, Faculty of Health and Medical Sciences, University of Copenhagen, Copenhagen, Denmark; 2 Exercise and Nutrition Research Program, Mary MacKillop Institute for Health Research, Australian Catholic University, Fitzroy, VIC 3000 Australia; 3 Center for Epigenetics and Metabolism, INSERM U1233, Department of Biological Chemistry, School of Medicine, University of California, Irvine, Irvine, CA USA; 4 Department of Molecular Medicine and Surgery, Karolinska Institutet, Stockholm, Sweden

**Keywords:** Metabolic disorders, Endocrinology

## Abstract

Time-restricted feeding (TRF) improves metabolism independent of dietary macronutrient composition or energy restriction. To elucidate mechanisms underpinning the effects of short-term TRF, we investigated skeletal muscle and serum metabolic and transcriptomic profiles from 11 men with overweight/obesity after TRF (8 h day^−1^) and extended feeding (EXF, 15 h day^−1^) in a randomised cross-over design (trial registration: ACTRN12617000165381). Here we show that muscle core clock gene expression was similar after both interventions. TRF increases the amplitude of oscillating muscle transcripts, but not muscle or serum metabolites. In muscle, TRF induces rhythmicity of several amino acid transporter genes and metabolites. In serum, lipids are the largest class of periodic metabolites, while the majority of phase-shifted metabolites are amino acid related. In conclusion, short-term TRF in overweight men affects the rhythmicity of serum and muscle metabolites and regulates the rhythmicity of genes controlling amino acid transport, without perturbing core clock gene expression.

## Abstract (web summary)

Time restricted feeding has several health benefits. Here the authors perform a randomised cross-over study with 11 men with overweight/obesity to investigate how time restricted feeding affects skeletal muscle and serum, and report that it does not affect the core circadian machinery, but modifies periodicity in amino acid related metabolites and transporters.

## Introduction

There is a growing appreciation that disrupted eating patterns, and a reduction in the time spent in the fasted state, lead to aberrant energy homeostasis that may predispose to chronic metabolic disorders^1,2^. In rodents, extending the duration of fasting improves glycemic control and reduces the incidence of cardiovascular disease^3^. Time-restricted feeding (TRF), typically defined as food consumed for <10 h per day, represents a practical means to control dietary intake by extending the time spent fasting and improves markers of metabolic health in both animal models^4–7^ and humans^8^. In rodents fed a high-fat diet, TRF attenuates body weight gain, reduces body fat accumulation, improves glucose tolerance, and restores diurnal rhythms of core clock transcripts compared to high-fat ad libitum diets^4,9^. Moreover, TRF protects against high-fat diet-induced obesity, fatty liver, dyslipidemia and glucose intolerance in mice with ablated circadian clock machinery^10^. In humans, time-restricted eating decreases body mass by reducing energy intake^11,12^, but improves whole-body insulin sensitivity and beta cell responsiveness independent of daily energy intake^8^. Collectively these results suggest that the interplay of energy intake timing (including time spent fasting), and whole-body circadian rhythmicity have a profound impact on whole-body metabolism and metabolic health. Analyses of the serum metabolome in humans and rodents have been performed under a variety of conditions^4,9,13,14^. However, the mechanisms underpinning the beneficial effects of TRF on metabolic health remain largely unknown.

We present the temporal relationship between circulating metabolites and corresponding skeletal muscle metabolite and gene transcript profiles using a short-term (5-day), controlled intervention of isoenergetic TRF (8 h day^−1^, 1000–1800 h) versus extended feeding (EXF; 15 h day^−1^, 0700–2200 h) using a crossover design in men with overweight/obesity. We observed that, the daily pattern of energy intake did not alter expression of the core clock machinery, TRF induced a greater number of periodic metabolites in serum compared to skeletal muscle, and TRF induced periodic expression of transcripts encoding fatty acid and amino acid transporters, coupled to the reciprocal regulation of amino acid metabolites in skeletal muscle and serum. Collectively, our results provide evidence demonstrating that short-term TRF in men with overweight/obesity affects periodic metabolism, while not influencing the expression of core clock genes.

## Results

### Participants

Eleven men (mean ± SD, age: 38 ± 5 years; body mass index: 32 ± 2 kg m^−2^; body mass: 103 ± 9 kg; body fat percentage: 34 ± 4%) completed both experimental conditions in a randomized, crossover design (Supplementary Fig. 1). The protocol and clinical characteristics of the cohort have been recently reported^15^.

### TRF affects periodicity of metabolites and transcripts

**Fig. 1:**
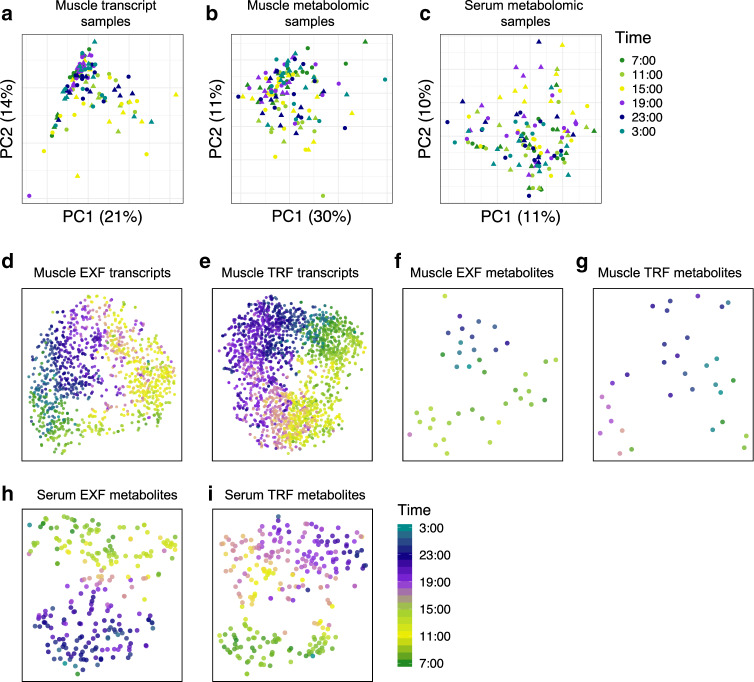
Dimensionality reduction of samples and periodic features. Principal component analysis (PCA) of samples based on skeletal muscle genes (**a**), skeletal muscle metabolites (**b**), and serum metabolites (**c**), with color indicating sampling time. Circle indicates extended feeding (EXF), and triangle indicates time-restricted feeding (TRF). t-SNE clustering of periodic transcripts in skeletal muscle after EXF (**d**), and TRF (**e**). Periodic metabolites in skeletal muscle after EXF (**f**), and TRF (**g**), and serum metabolites after EXF (**h**), and TRF (**i**). *n* = 11 participants.

Principal component analysis (PCA) of skeletal muscle transcripts (Fig. 1a) and metabolites (Fig. 1b), and serum metabolites (Fig. 1c) did not separate samples by either time or intervention. Conversely, t-Distributed Stochastic Neighbor Embedding (t-SNE) clustering of skeletal muscle transcripts after EXF and TRF (Fig. 1d, e) showed clear clustering based on acrophase. Clustering of skeletal muscle metabolites was less clear with both feeding protocols, (Fig. 1f, g), while serum metabolites showed clear clustering based on acrophase after EXF and TRF (Fig. 1h, i).

### Overlaps of periodic metabolites and transcripts

**Fig. 2:**
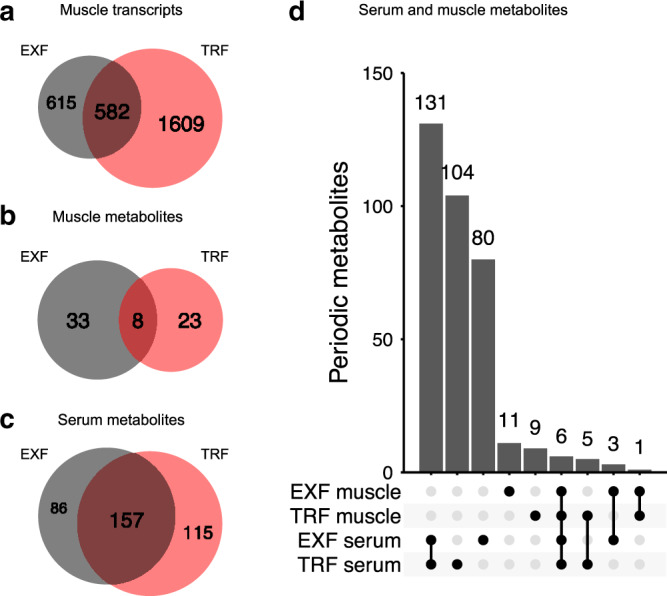
Overlaps of significantly periodic features in serum and skeletal muscle. Periodic skeletal muscle transcripts (**a**), skeletal muscle metabolites (**b**), and serum metabolites after extended feeding (EXF) in gray (**c**), and time-restricted feeding (TRF) in light red. Comparison of periodic serum and skeletal muscle metabolites (with only relevant comparisons shown) (**d**). Vertical bars indicate set size, dots and lines indicate set identity, *n* = 11 participants.

The proportion of periodic features varied widely for each condition, with both feeding protocols inducing oscillations with a 24 h period in a small proportion (8% EXF and 15% TRF, respectively) of skeletal muscle transcripts measured (14,954 transcripts), and 8% in either condition having a period of 12 h (Supplementary Fig. 2). We identified 1,609 oscillating skeletal muscle transcripts unique to TRF, and 615 unique to EXF, with 582 shared between the two conditions (Fig. 2a). Out of the 493 skeletal muscle metabolites measured, 7 and 4% of the metabolites were oscillating, with a period of 24 h, and 3 and 4% with a period of 12 h after EXF and TRF, respectively (Supplementary Fig. 2). TRF induced 33 unique periodic metabolites, while EXF induced 23 unique periodic metabolites in skeletal muscle, with 8 shared between the two conditions (Fig. 2b). Analysis of the serum revealed a total of 775 serum metabolites, with 31% oscillating metabolites identified after EXF, and 35% after TRF with a period of 24 h, and 3 and 1% with a period of 12 h after EXF and TRF, respectively (Supplementary Fig. 2). We found 115 and 86 metabolites were uniquely periodic after TRF and EXF respectively, with 157 metabolites shared between the two conditions (Fig. 2c). Comparing the periodic metabolites in skeletal muscle and serum revealed that the biggest group (131 metabolites) were shared in the serum between EXF and TRF, with 6 common metabolites across the feeding protocols in both skeletal muscle and serum (Fig. 2d). When comparing skeletal muscle and serum, we found 3 unique metabolites after EXF, and 5 unique metabolites after TRF (Fig. 2d). A list identifying significant transcripts and metabolites, along with associated *p*-values, acrophase, amplitude and MESOR is reported (Supplementary Data 1).

### Rhythm characteristics of periodic features

**Fig. 3:**
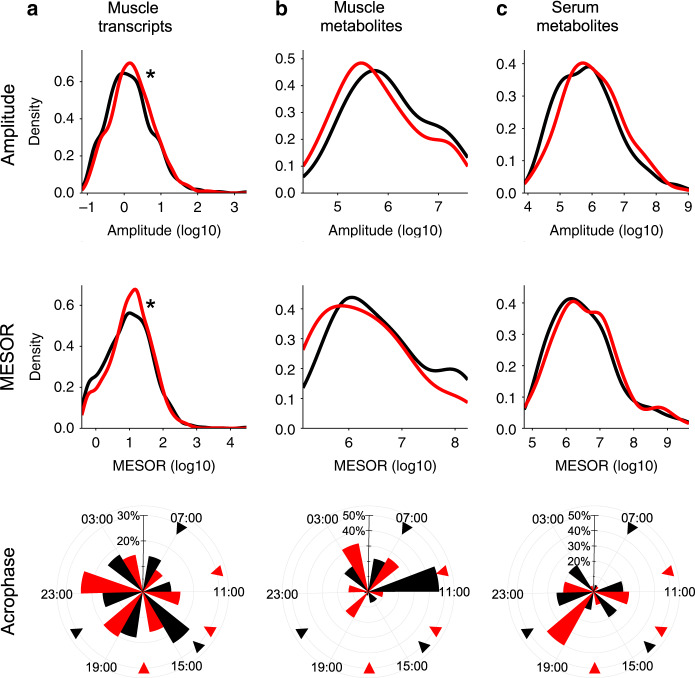
Amplitude and peak distribution of periodic features. Density plots of significantly periodic features in each respective condition, showing amplitude, MESOR and acrophase with black line representing extended feeding (EXF), and red line representing time-restricted feeding (TRF), for skeletal muscle transcripts (**a**), skeletal muscle metabolites (p_mesor_ = 0.0018, and p_amplitude_ = 0.0006) (**b**), and serum metabolites (**c**). Triangle indicates feeding time. *p < 0.05 Kolmogorov–Smirnov two sample and two sided test for EXF versus TRF, *n* = 11 participants.

Skeletal muscle transcripts showed a small but significant difference for a higher amplitude and MESOR after TRF. While the overall pattern of acrophase distribution was comparable between the feeding protocols, most transcripts peaked at 1500 h for EXF, and 2300 h for TRF (Fig. 3a). Skeletal muscle metabolites had a similar distribution of amplitude and MESOR, with the majority of metabolites peaking at 1100 h for EXF and 0300 h for TRF (Fig. 3b). Serum metabolites showed no difference in the amplitude or MESOR distribution, with the peak times showing a bimodal distribution after EXF and TRF (Fig. 3c).

### Metabolite circadian misalignment

**Fig. 4:**
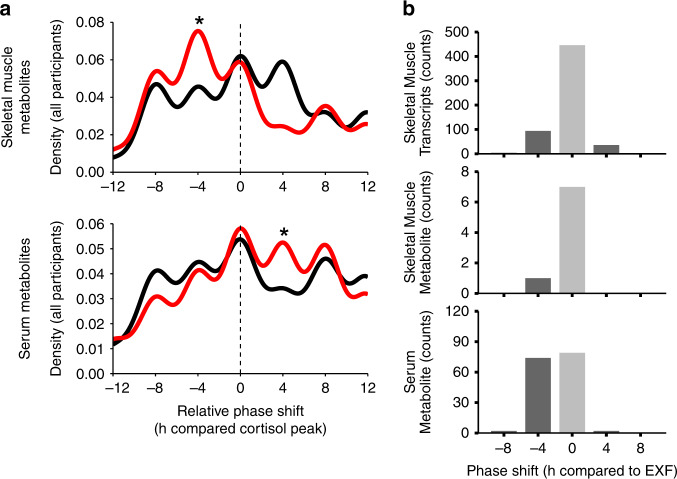
Circadian alignment of periodic features. Density plot showing the relative phase shift compared to the cortisol maximum for all individuals combined for skeletal muscle (top panel) and serum metabolites (bottom panel) with the black line showing extended feeding (EXF), and the red line showing time-restricted feeding (TRF) (**a**). Histogram of TRF phase shift relative to EXF at the feature level for skeletal muscle transcripts (top panel, *p* < 2.2 * 10^−16^), skeletal muscle metabolites (middle panel) and serum metabolites (bottom panel. *p* ≈ 7.74 * 10^−12^) (**b**). *p < 0.05 Kolmogorov–Smirnov two sample and two sided test for EXF versus TRF, *n* = 11 participants.

The relative circadian alignment (phase adjusted to cortisol) of each participant indicated that TRF induced a phase advance in skeletal muscle metabolites as compared to EXF, with opposite and smaller differences observed in serum (Fig. 4a). When comparing the acrophase of skeletal muscle transcripts or metabolites between the feeding protocols, the majority of these features had a similar peak time. However, a small subset of skeletal muscle transcripts and metabolites had a phase advance of 4 h in TRF versus EXF. Conversely, when comparing the serum metabolites, approximately equal numbers of features showed an unchanged peak time or a phase advance of 4 h in TRF versus EXF (Fig. 4b).

### Core clock gene oscillations in skeletal muscle

**Fig. 5:**
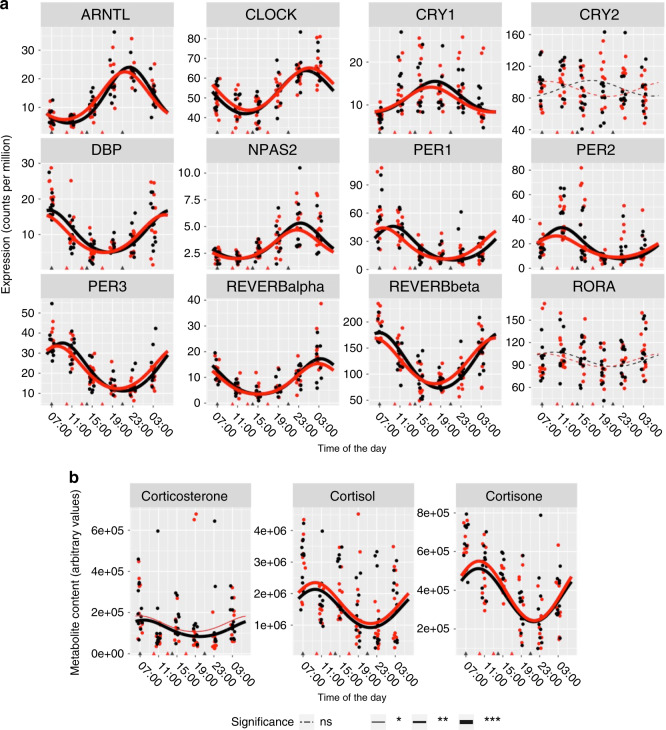
Periodicity of clock machinery. Expression of core clock genes in skeletal muscle (**a**) and corticosteroids in serum (**b**). Black line color indicates extended feeding (EXF), and red line color indicates time-restricted feeding (TRF). Points are individual datapoints, and lines represent cosinor regression fit. Triangle indicates feeding time, line type indicates FDR adjusted, RAIN derived *p* value for either EXF, or TRF; ns *p* > 0.05, *p < 0.05, **p < 0.01, ***p < 0.001, *n* = 11 participants.

Skeletal muscle expression of core clock genes *ARNTL*, *CLOCK*, *CRY1*, *DBP*, *NPAS2*, *REVERB alpha*, *REVERB beta*, *PER1*, *PER2,* and *PER3* exhibited periodic oscillations after both feeding protocols, while *CRY2* and *RORA* did not show periodic oscillations in response to either intervention (Fig. 5a). There were no significant differences between the feeding protocols for either MESOR, amplitude or acrophase for any of the core clock genes. Corticosterone, cortisol and cortisone showed significant periodicity after both feeding protocols, with no significant differences in either MESOR, amplitude or acrophase (Fig. 5b).

### Functional enrichment of periodic features

**Fig. 6:**
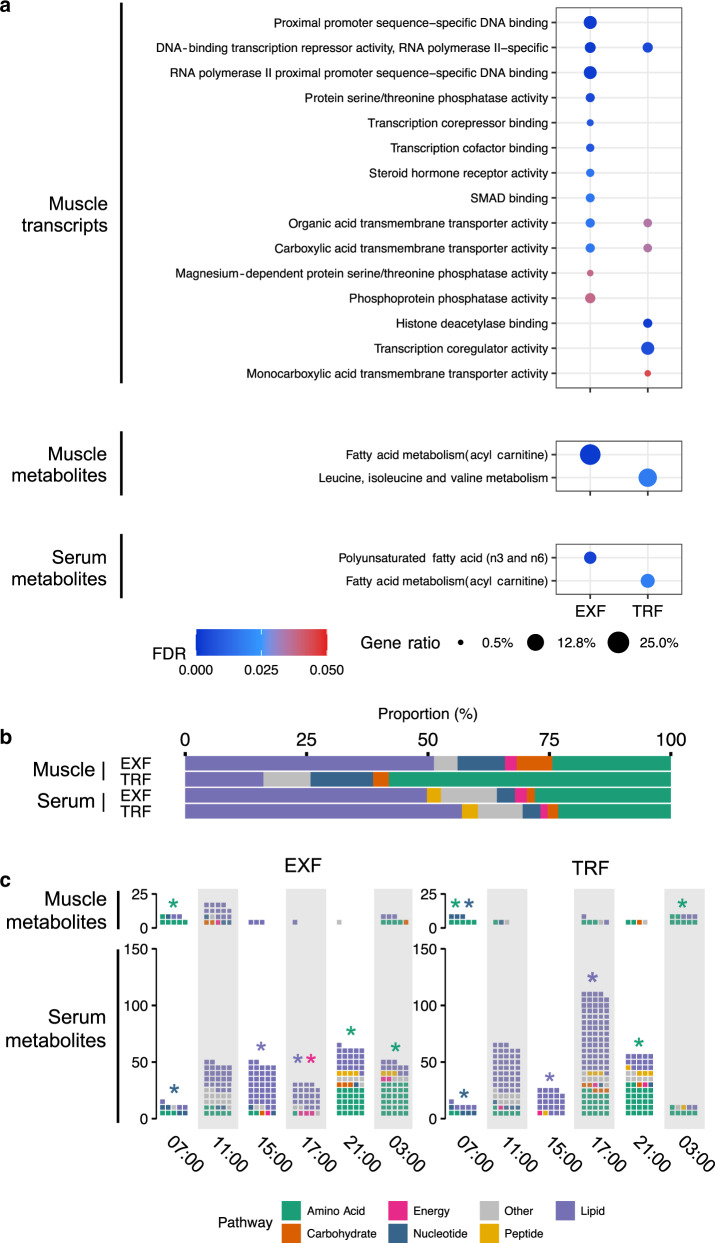
Functional enrichment of periodic features. Over-representation analysis of molecular function gene ontology in skeletal muscle periodic transcripts (top panel), and Metabolon sub-pathway definition of skeletal muscle metabolites (middle panel), and serum metabolites (bottom panel) after unrestricted feeding (EXF) and time-restricted feeding (TRF). Color indicates FDR adjusted *p* value; circle size indicates the proportion of periodic genes in the ontology (**a**). Proportion of Metabolon super-pathway definition of periodic metabolites in skeletal muscle and serum after EXF and TRF (**b**). Counts of super-pathway definition of periodic metabolites in skeletal muscle and serum after EXF and TRF at each measured timepoint. Color indicates metabolite classification (**c**). *p < 0.05, *n* = 11 participants.

Gene ontology enrichment analysis of periodic genes in skeletal muscle indicated that both feeding protocols induced periodic oscillations in genes encoding regulators of transcriptional activity. EXF induced oscillations of genes encoding regulation of transcription factor activity and protein phosphatase activity, while TRF induced oscillations of genes encoding histone deacetylation activity, transcriptional regulation and monocarboxylic acid transporter activity. Both EXF and TRF showed enrichment of genes associated with organic acid and carboxylic acid transmembrane transporter activity (Fig. 6a). Skeletal muscle metabolites were enriched for fatty acid metabolism after EXF, whereas metabolites for leucine, isoleucine and valine metabolism were enriched after TRF (Fig. 6a). Serum metabolites were enriched for polyunsaturated fatty acids after EXF and fatty acid metabolism after TRF (Fig. 6a). Ultradian 12 h periodic transcripts were enriched for 1-phosphoinositol-3 kinase, receptor, and transcription activity in both EXF and TRF (Supplementary Fig. 3a). Ultradian 12 h periodic transcripts were enriched for carbohydrate binding and metalloendopeptidase activity in EXF, and collagen binding, calcium ion binding, low-density lipoprotein particle binding and peptide binding (among other) in TRF (Supplementary Fig. 3a). Ultradian skeletal muscle metabolites were enriched for ceramides, as well as leucine, isoleucine and valine metabolism after EXF, and ceramides and diacylglycerols after TRF (Supplementary Fig. 3a). We also found that 12 h period serum metabolites were enriched for leucine, isoleucine and valine metabolism, as well as gamma-glutamyl amino acids after both EXF and TRF (Supplementary Fig. 3a). Lipids were the largest class of periodic metabolites in serum after either EXF or TRF (Fig. 6b), while the predominant class of skeletal muscle metabolites were lipids and amino acids after EXF and TRF, respectively (Fig. 6b). The distribution of skeletal muscle and serum metabolites over the daily cycle showed a large variation. There was a significant enrichment in skeletal muscle metabolites related to amino acid metabolism at 0700 h after EXF, as well as at 0700 and 0300 h after TRF, and an enrichment of nucleotide-related metabolites at 0700 h after TRF. Serum showed an enrichment for lipid-related metabolites at 1500 and 1700 h, and for nucleotide-related metabolites at 0700 h after both feeding protocols. Amino acid-related metabolites were enriched at 2100 h for both feeding protocols, as well as at 0300 h after EXF. Energy-related metabolites were enriched at 1700 h after EXF (Fig. 6c). The majority of the unique skeletal muscle metabolites identified in response to each feeding protocol were lipid-related after EXF, and amino acid-related after TRF (Supplementary Fig. 3b).

### Differences in periodicity between EXF and TRF

**Fig. 7:**
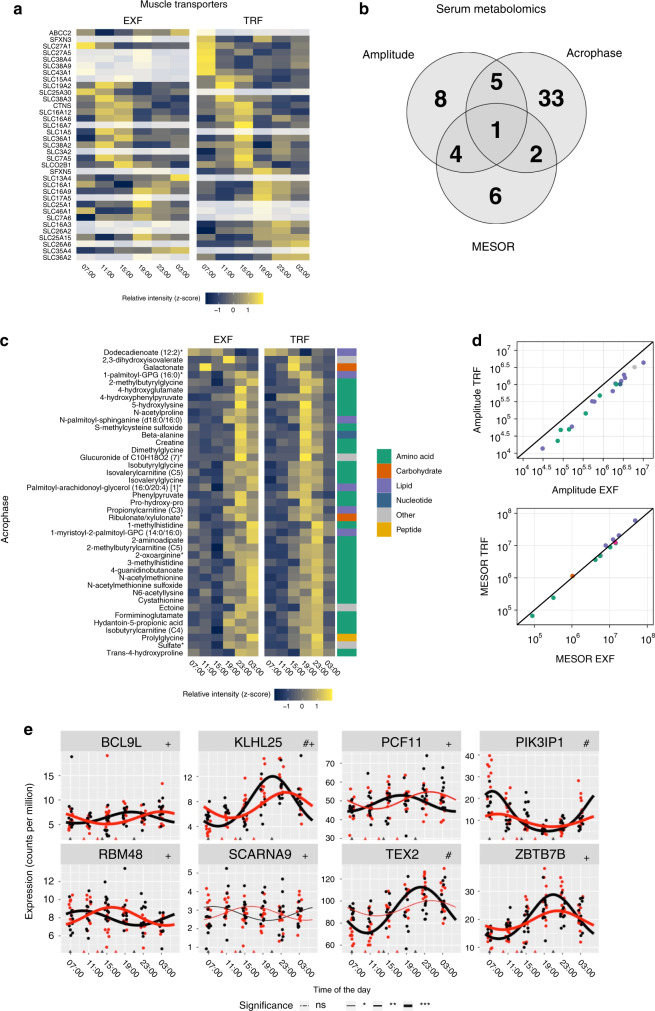
Transporter periodicity, and differential transcript and serum metabolite rhythmicity. Heatmap of gene ontology transporter annotated genes enriched in either extended feeding (EXF) or time-restricted feeding (TRF), grayed out cells indicate non-significant rhythmicity, and color *z*-score normalized expression (**a**). Serum metabolite significant differences in acrophase, amplitude, or MESOR (**b**). Heatmap of serum metabolites with differential acrophase between EXF and TRF; cell color indicates *z*-score normalized expression, and Metabolon super-pathway annotation is indicated in color to the right. (**c**). Scatterplot of serum metabolites with significant differences in amplitude, with amplitude of EXF on the *x*-axis, and TRF on the *y*-axis (top panel), and serum metabolites with differential MESOR, with EXF on the *x*-axis, and TRF on the *y*-axis (bottom panel), colors indicate super-pathway annotation (**d**). Expression of genes with significant differences in FDR adjusted *p* value in either amplitude (#) or acrophase (+) derived from CircaCompare. Points are individual datapoints, and line represents cosinor regression fit. Black line indicates EXF, and red line indicates TRF. Triangles on the horizontal axis indicate feeding time, line type indicates FDR adjusted RAIN derived *p* value (**e**); ns *p* > 0.05, *p < 0.05, **p < 0.01, ***p < 0.001. *n* = 11 participants.

Both EXF and TRF induced rhythmicity in genes encoding various transporters, with TRF inducing rhythmicity in several amino acid transporters (Fig. 7a). Differential analysis with respect to MESOR, amplitude, and acrophase, showed the majority of differential serum metabolites were altered with respect to acrophase, with 33 metabolites differing only in acrophase, six only in MESOR, and eight only in amplitude (Fig. 7b). The majority of serum metabolites with differential acrophase or MESOR were amino acid-related, while most metabolites with differential amplitude were lipid-related (Fig. 7c-d). EXF induced consistently higher amplitudes of serum metabolites, and higher MESOR of amino acid- and lower MESOR of lipid-related metabolites, (Fig. 7d). Differentially rhythmic genes were involved in RNA processing and PI3K regulation, with most of the genes having differential acrophase and amplitude, but not MESOR (Fig. 7e). We did not identify any dietary-induced skeletal muscle metabolites in either MESOR, amplitude or acrophase.

## Discussion

Circadian regulation of transcriptional processes impact cellular metabolism and homeostasis, with the timing and composition of energy intake profoundly influencing diurnal rhythms in peripheral tissues^4,14,16–20^. Precisely how nutritional challenges are differentially interpreted by distinct tissue-specific clocks, remains largely unexplored. Here, we interrogated the skeletal muscle transcriptome, and serum and skeletal muscle metabolome of men with overweight/obesity, to determine how restricting the window of food intake (from 15 to 8 h day^−1^) confers some of the metabolic health benefits that have been reported after TRF^8,12^. In this cohort, short-term TRF reduced nocturnal glucose levels and improved insulin profiles throughout the day^15^. Serial sampling of serum and skeletal muscle over a 24-h period provides a comparative analysis of the diurnal metabolome in serum versus skeletal muscle in humans and is critical to decipher those circulating metabolites that constitute specific metabolic signatures of differential nutritional challenges. This approach also provides insight into how meal-timing influences rhythmicity of tissue-specific clocks. We demonstrate that TRF affects the rhythmicity of serum and skeletal muscle metabolites, and underpins the rhythmicity of amino acid transporter genes, as well as amino acid and lipid metabolites, without perturbing the expression of core clock genes.

PCA revealed no clear clustering of samples either by feeding protocol or time-of-sampling, implying that the observed systematic biological variance was not dominated by coordinated periodic variation in gene expression or metabolite content. This suggests that TRF has discreet effects on human metabolism, a finding supported by the small overall percentage of periodic skeletal muscle transcripts and metabolites, as well as the similarity of the serum metabolite signature induced by either EXF or TRF. Conversely, similarity analysis of skeletal muscle transcripts and skeletal muscle and serum metabolites showed clear clustering based on time of peak, indicating that the calculated acrophase of periodic transcripts and metabolites predicts their oscillatory pattern.

A basic paradigm of circadian regulation of metabolism is that oscillations of gene expression generate daily rhythms in cellular metabolism^21^. However, not all periodic oscillations are under the control of the core clock machinery. For example, metabolic activity of red blood cells can be periodic, independent of the core clock machinery^22^. Moreover, glucose homeostasis is improved by TRF in high-fat fed obese mice lacking a circadian clock^10^, although this latter find requires confirmation in other tissue-specific clock-deficient mouse models. Nevertheless, such observations highlight differences in the regulation and adaptation to diurnal variation in various cells and organs. In humans, the expression of core clock machinery is modulated by TRF in whole blood cells or leukocytes^23,24^. Breakfast skipping adversely affects clock-controlled gene expression in leukocytes, concomitant with increased postprandial glycemia in both healthy individuals and patients with type 2 diabetes^23^. Early TRF, whereby all daily food intake is consumed by 1500 h, improves whole-body glucose and lipid metabolism and shifts circadian clock gene expression in whole blood^24^. While these studies indicate that meal timing influences circadian gene expression in whole blood cells or leukocytes, they lack a complementary analysis of clock gene expression in peripheral tissues with roles in whole-body metabolic regulation. Emerging evidence highlights a variety tissue-specific transcriptomic and metabolomic profiles^25^, emphasizing the need to combine data from more than one tissue to decipher signatures associated with responses to nutritional challenges. This is particularly important when extrapolating results of gene expression of whole blood or leukocytes to peripheral organs involved in glucose homeostasis.

Under a variety of metabolic insults, the consistency of the core clock gene oscillations demonstrates the remarkable resilience of the circadian clock time keeping system^2,21^. However, the metabolic clock output genes (some of which are transcriptional regulators) often show more profound alterations, with loss of gain of oscillations or phase and amplitude changes^2,21^. We identified large differences in the number periodic transcripts with both feeding protocols, consistent with the overall number of circadian transcripts from a previous report in humans^26^, but without differences in the expression of core clock genes in skeletal muscle, or the circadian hormones^27,28^, cortisol, cortisone and corticosterone. In rodents, TRF increases the amplitude of rhythms and expression of circadian clock genes and clock output genes in liver, with a greater effect on liver metabolites in high-fat fed versus chow fed mice^4^. This dramatic effect of TRF on core clock genes in high-fat fed mice was associated with marked effects on body weight, hepatosteatosis, inflammation, and glucose metabolism^4^. There are several possible explanations for the discrepancies between these observations, including duration of TRF, extent of obesity, species or tissues studied. A recent study in mice indicates that rhythmic food intake drives rhythmic gene expression independently of the intrinsic autonomous circadian clock in liver^29^, indicating food cues, possibly interacting with the master clock in the suprachiasmatic nucleus^30^, synchronize the circadian programs of peripheral clocks. Skeletal muscle displays robust core clock rhythmicity^31^, even under circadian light challenge^32^, but whether systemic control by energy intake can offset the tissue-specific intrinsic clock machinery is unknown. Our data suggest that metabolic circadian output genes and metabolite responsive genes, rather than core clock genes are altered by feeding behavior. We found that the total number of skeletal muscle periodic metabolites was modest, with ~20–25% similarity between both feeding protocols. Moreover, we found that more serum metabolites and skeletal muscle genes were periodic after TRF, suggesting that in the short-term, postprandial meal-timing effects may play a role in regulating diurnal variation of metabolism.

We found that TRF increased the amplitude of skeletal muscle transcripts, but not skeletal muscle or serum metabolites. Conversely, we found that the majority of differential periodic serum metabolites had decreased amplitudes. The amplitude of periodic metabolites in serum is unaffected in men with type 2 diabetes^33^, or in serum and skeletal muscle of high-fat fed rodents^25^, whereas the amplitude of periodic metabolites is reduced in serum of healthy young men subjected to 24 h of wakefulness^34^. These data, together with our finding of unchanged core clock machinery, suggest that TRF can independently modulate specific aspects of circulatory and skeletal muscle metabolic circadianicity distinct from pathophysiological states such as type 2 diabetes or obesity. Thus, food cues may play a regulatory role on the metabolomic profile, in addition to regulation via the clock output genes. We detected a bimodal distribution across the daily cycle only for serum metabolites after EXF, and a lack of an apparent similarity of the time of peaks for all other features. The difference in the acrophase of a feature between the feeding protocols indicates that metabolites with different peak time are consistently phase-advanced in TRF as compared to EXF. We found that skeletal muscle had a larger proportion of phase-advanced transcripts, but also some phase-delayed transcripts. In addition, the relative phase (cortisol adjusted time per individual), differed between serum and skeletal muscle metabolites. Collectively, our observations highlight divergence between the skeletal muscle transcriptome, the skeletal muscle metabolome, and the serum metabolome in response to different feeding protocols. In serum many changes are related to postprandial meal-timing effects, whereas in skeletal muscle, given the similar peak times in many features, not all the effects can be attributed to the postprandial state. Future functional studies using labeled metabolites may resolve this discrepancy.

The periodic transcripts and metabolites common between TRF and EXF could represent fundamental metabolic processes. Indeed, skeletal muscle gene ontology over-representation analysis showed enrichment for transcriptional regulation after both feeding protocols. Transcripts with a 12 h circadian period showed an enrichment for transcriptional activity, and for PI3K regulatory activity. Additionally, transcripts with differential rhythmicity were involved in RNA processing^35–37^ and transcriptional regulation^38–40^, as well as PI3K/AKT signaling^41^. These findings corroborate results from rodent studies showing that fasted circadian sampling regulates skeletal and cardiac muscle anabolic signaling^27^. Transporter activity-related gene ontology terms were enriched after both TRF and EXF, but with a different subset of genes orchestrating the enrichment. Of note, several amino acid transporters were periodic only after TRF. Skeletal muscle metabolites were enriched for amino acid annotated metabolites after TRF, but not EXF, while serum metabolites were enriched for lipid metabolism after both feeding protocols. Lipid metabolites account for ~80% of the metabolites with entrained circadian oscillations^13^, while diurnal disturbances alter fatty acid metabolism^32^, and TRF for 5 weeks increases serum triglycerides^8^. We found that the majority of the periodic serum metabolites detected after either TRF or EXF were lipids. However, the metabolites with significant acrophase differences between EXF and TRF, and the majority of skeletal muscle TRF-specific metabolites, were mostly amino acid-related. Collectively, our results provide evidence to suggest that TRF induces a time-of-day specific response on the metabolome, involving increased uptake of amino acids from the serum, and increased amino acid metabolism in skeletal muscle. The phases of the features shared between the diets were either unchanged, or phase advanced, suggesting that the timing of food intake plays a role in reprogramming the diurnal control of metabolism.

There are several strengths and limitations of the current study. While several studies have investigated the immediate postprandial profile of serum metabolomics^42–46^, our study investigated free living, daily rhythms, and has potential translational outcomes that may be clinically relevant. In addition, we also moved beyond serum metabolite profiling and assessed the effect of different feeding protocols on the metabolite and transcriptomic profile of skeletal muscle, a peripheral tissue important for whole-body metabolic regulation. Our sample size was ameliorated by our crossover design, and included a 4-day harmonization period, during which the participants were monitored for dietary compliance, thus minimizing the confounding effects of individual variation. Of note, the study design was not intended to capture entrained circadian metabolites and transcripts. Rather, the intention was to identify ecologically valid diurnal metabolites and transcripts. While an untargeted metabolite analysis was performed, the large diversity of the human metabolome could not be fully captured in our analysis due to technical limitations related to the fact that the identity of most features detected remain unknown. Future studies with broader metabolite profiling will enable efforts to characterize the diurnal response of an organism to metabolic perturbations or disease pathogenesis.

In conclusion, we have used transcriptomics and untargeted metabolomics to characterize both serum and skeletal metabolic signatures in response to a controlled intervention of isoenergetic time restricted versus extended feeding. We provide evidence to suggest that short-term TRF modulates the diurnal rhythm of lipid and amino acid metabolism, without modulating the expression of core clock genes in skeletal muscle. Long-term studies of time restricted versus extended feeding in humans in real world settings, employing targeted interrogative molecular techniques are required to determine the precise mechanisms underlying the previously observed health-related benefits of a time restricted regimen.

## Methods

### Experimental subject details

11 men (aged 30–45 years) with overweight/obesity (body mass index [BMI] 27–35 kg m^−2^) following a sedentary lifestyle (<150 min wk^−1^ exercise and >3 h d^−1^ sitting) were recruited to participate. Sample size was choosen based on previous published research^14,31^. Clinical characteristics of the study participants have been previously reported^15^. First enrollment of participants was on 30/01/2017, and trial was completed by 22/06/2017. Measures of insulin sensitivity (i.e. oral glucose tolerance or euglycemic hyperinsulinemic clamp tests) were not performed in this cohort. The study was approved by the Human Research Ethics Committee of the Australian Catholic University (2016-215H) and informed written informed consent was obtained from each participant.

### Dietary intervention and biopsy procedure

The study employed two experimental dietary conditions whereby the participants consumed prepared meals consisting of ~32% of total energy intake (TEI) from carbohydrate, ~49% TEI from fat, and ~19% of TEI from protein for five days. Participants were randomized to start either EXF or TRF using computer generated random numbers placed in sealed opaque envelopes (block-randomization, *n* = 4), and were revealed to laboratory personel after completion of baseline measurements.  After this, the investigators were not blinded for group allocation. The timing of meals differed between the two conditions, where energy was either consumed between a 15 h “extended” feeding (EXF) window of 0700 to 2200 h or an 8 h “time-restricted” feeding (TRF) window of 1000–1800 h. Participants consumed the meals at standardized times within ±30 min (at 0700, 1400 and 2100 h for EXF and at 1000, 1300, and 1700 h for TRF) throughout both experimental conditions. On the fifth day of the experimental conditions, participants arrived at the laboratory at ~0630 h after an overnight fast and remained in the laboratory until 0730 h the following day (i.e. for a 24 h period) in order to obtain a total of six skeletal muscle biopsies and blood samples every 4 h (Supplementary Fig. 1). Participants were free-living in the laboratory, aside from a structured walk period (~700 m) 1 h after each meal (i.e. 3 × day). At ~2230 h participants were taken to the Nursing Simulation Suite at ACU for the overnight portion of the 24-h condition period.

Throughout the 24 h laboratory visit, *vastus lateralis* muscle biopsies (~150-300 mg) were obtained every 4 h (from 0700 h) using local anesthesia (2–3 mL of 1% Xylocaine (lignocaine)) and a 6 mm Bergstrom needle modified with suction, and were immediately frozen using liquid nitrogen before being stored at −80 °C for later analysis. The participants were in natural light during the day and slept in a darkened room during the night. Muscle biopsies and blood samples were taken without turning on the lights, with the doctor using a miniature head-mounted portable light source as the only illumination. An in-dwelling venous catheter was inserted and blood (5 mL Serum clot activator, Greiner Bio-One) was collected prior to each muscle biopsy while participants were supine. Throughout the 24 h sampling period, cannulas were kept patent with regular saline (0.9% NaCl) flushes. The serum samples remained at room temperature for 30 min before centrifuging at 3000 *g*, for 10 min at 4 °C and was then aliquoted and stored at −80 °C for subsequent analysis.

### Metabolomic and transcriptomic analysis

Metabolomics analysis was performed by Metabolon Inc. (Durham, NC), as described^47^. Briefly, small biochemicals from skeletal muscle and serum were methanol extracted and analyzed by ultra-high-performance liquid chromatography-tandem mass spectrometry (UPLC-MS/MS; positive mode), UPLC-MS/MS (negative mode) and gas chromatography-MS (GC-MS). The UPLC-MS/MS platform utilized a Waters Acquity UPLC with Waters UPLC BEH C18-2.1 × 100 mm, 1.7 μm columns and a ThermoFisher LTQ MS, which included an electrospray ionization source and a linear ion-trap mass analyzer. Samples destined for analysis by gas chromatography mass spectrometry (GC-MS) were dried under vacuum desiccation for a minimum of 18 h prior to being derivatized using bis(trimethylsilyl)trifluoroacetamide. Derivatized samples were separated on a 5% phenyldimethyl silicone column with helium as carrier gas and a temperature ramp from 60 °C to 340 °C within a 17-min period. All samples were analyzed on a Thermo-Finnigan Trace DSQ fast-scanning single-quadrupole MS operated at unit mass resolving power with electron impact ionization and a 50–750 atomic mass unit scan range. Metabolites were identified by automated comparison of the ion features in the experimental samples to a reference library of chemical standard entries that included retention time, molecular weight (m/z), preferred adducts, and in-source fragments as well as associated MS spectra and were curated by visual inspection for quality control using software developed at Metabolon, Inc^48^. Missing data were imputed using k-nearest neighbor imputation. Raw signal intensity of all metabolites detected are presented in Supplementary Data 2.

RNA quality was assed using the Bioanalyzer instrument (Agilent Technologies) and RNA sequencing libraries were prepared using the Illumina TruSeq Stranded Total RNA with Ribo-Zero Gold protocol (Illumina). Libraries were sequenced on a NextSeq500 instrument (Illumina) with 38- bp paired end. Reads were mapped to ENSEMBL hg38 release 92 using STAR aligner^49^, and transcripts were counted with FeatureCounts^50^ using gencode release 27. Sequencing depth ranged from 52.9 to 10.4 million with an average of 20.5 million reads. Reads were filtered using filterbyExpression^51^, with minimum count of 10. Transcriptomic data is deposited under accession number GSE129843.

### Quantification and statistical analysis

Periodic features in each feeding protocol were identified using the non-parametric RAIN algorithm^52^, with an independent method (to avoid false positive increasing trends), and delta.period of 12 allowing for the detection of features with a period of 12–28 h. Rhythmic features were defined as any feature with a Benjamini–Hochberg false discovery rate adjusted *p* value (FDR) < 0.05, and periods of 12 or 24. Amplitude, MESOR, and acrophase were detected using the non-linear cosinor regression tool CircaCompare^53^, using *Y* = *k* +  ⍺cos[τ(*t* − *φ*)] and the period defined by RAIN. Because many features violated assumptions for cosinor regression, we power transformed the data using the lambda calculated from boxcoxfit, unless lambda was 0, or greater or smaller than 2, where we log10 transformed the data. The FDR obtained by RAIN was considered as the *p* value of rhythmicity for the feeding protocols, and comparisons in acrophase, MESOR and amplitude between EXF and TRF were only investigated in features with significant FDR after both feeding protocols, using CircaCompare. A schematic of the bioinformatic pipeline is presented in supplementary Fig. 4. Amplitudes and MESORs were then back transformed using lambda, and for subsequent analysis. Density plot comparisons were performed using two tailed Kolmogorov–Smirnov test. Circadian misalignment was calculated by taking the timepoint with the maximal value of cortisol and subtracting the timepoint of the maximal value for each participant. Gene ontology molecular function enrichment, and metabolite class enrichment using Metabolon super-pathway and sub-pathway definitions as sets, was performed using compareClucter function from ClusterProfiler with default settings^54^. t-SNE analysis was performed using Rtsne package, with perplexity of 30 for skeletal muscle gene expression and serum metabolite content, and perplexity of 10 for skeletal muscle metabolite content. PCA analysis was performed on *z*-score transformed using the prcomp function. Following recruitment, no samples or features were excluded from the analysis.

### Ethics declarations

The study was approved by the ACU Human Research Ethics Committee (2016-215H).

### Reporting summary

Further information on research design is available in the Nature Research Reporting Summary linked to this article.

## Supplementary information

Supplementary Information

Description of Additional SupplementaryFiles

Supplementary Data 1

Supplementary Data 2

Reporting Summary

## Data Availability

Transcriptomic data are made available in the Gene Expression Omnibus Depository under the accession code GSE129843. Raw serum and skeletal muscle metabolomics data are not available. Serum and skeletal muscle metabolomics processed data are provided with in Supplementary Data 2. Source data are provided with this paper.

## Source data

Source Data

## Code availability

All scripts used to analyze feature rhythmicity and generate figures can be found at https://github.com/leonidaslundell/GSE129843.
