# Author Correction: Time-restricted feeding alters lipid and amino acid metabolite rhythmicity without perturbing clock gene expression

**DOI:** 10.1038/s41467-020-19100-5

**Published:** 2020-10-08

**Authors:** Leonidas S. Lundell, Evelyn B. Parr, Brooke L. Devlin, Lars R. Ingerslev, Ali Altıntaş, Shogo Sato, Paolo Sassone-Corsi, Romain Barrès, Juleen R. Zierath, John A. Hawley

**Affiliations:** 1 Novo Nordisk Foundation Center for Basic Metabolic Research, Faculty of Health and Medical Sciences, University of Copenhagen, Copenhagen, Denmark; 2 Exercise and Nutrition Research Program, Mary MacKillop Institute for Health Research, Australian Catholic University, Fitzroy, VIC 3000 Australia; 3 Center for Epigenetics and Metabolism, INSERM U1233, Department of Biological Chemistry, School of Medicine, University of California, Irvine, Irvine, CA USA; 4 Department of Molecular Medicine and Surgery, Karolinska Institutet, Stockholm, Sweden

**Keywords:** Metabolic disorders, Endocrinology

Correction to: *Nature Communications* 10.1038/s41467-020-18412-w, published online 16 September 2020.

The original version of this Article contained an error in the author affiliations.

Juleen R. Zierath was incorrectly associated with ‘Center for Epigenetics and Metabolism, INSERM U1233, Department of Biological Chemistry, School of Medicine, University of California, Irvine, Irvine, CA, USA’

This has now been corrected in both the PDF and HTML versions of the Article.

