# Supplementary material for: Time-restricted feeding alters lipid and amino acid metabolite rhythmicity without perturbing clock gene expression
Source: Nat Commun. 2020 Sep 16;11:4643. doi: 10.1038/s41467-020-18412-w (PMC7495469; doi:10.1038/s41467-020-18412-w)
Supplement: Supplementary file 1 — Supplementary Information [file 41467_2020_18412_MOESM1_ESM.pdf]

Time-Restricted Feeding Alters Lipid and Amino Acid Metabolite Rhythmicity without Perturbing Clock Gene Expression

Lundell *et al.*

Supplementary information

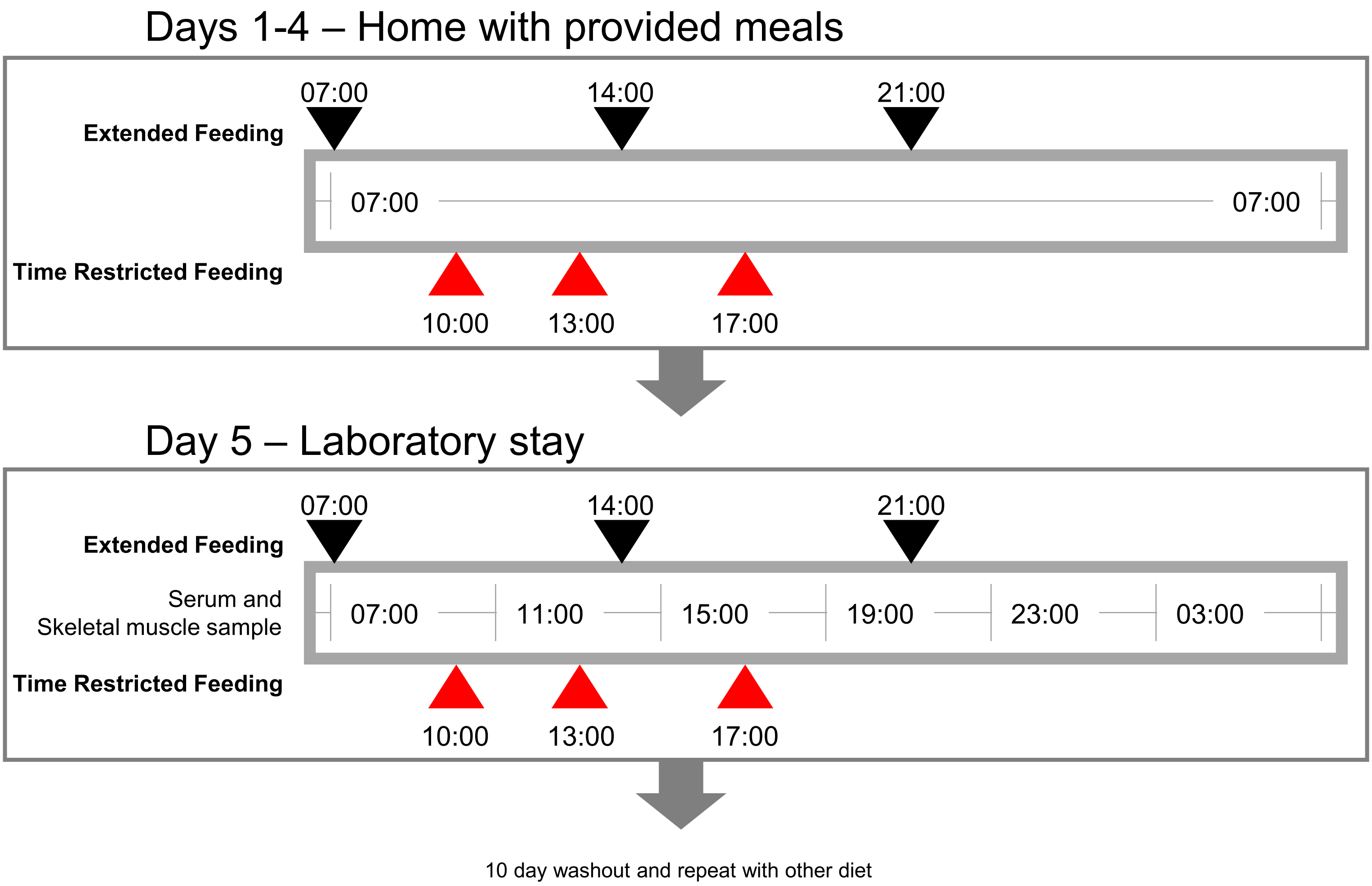

Supplementary Figure 1. Schematic overview of experimental design. Overall intervention for either extended feeding (EXF), or time-restricted feeding (TRF). Participants consumed provided diets in the appropriate schedule. Black triangle indicates EXF feeding times, and red triangle indicates TRF feeding times. Crossover to the respective diet was preceded by a 10-day washout period.

Supplementary Figure 2

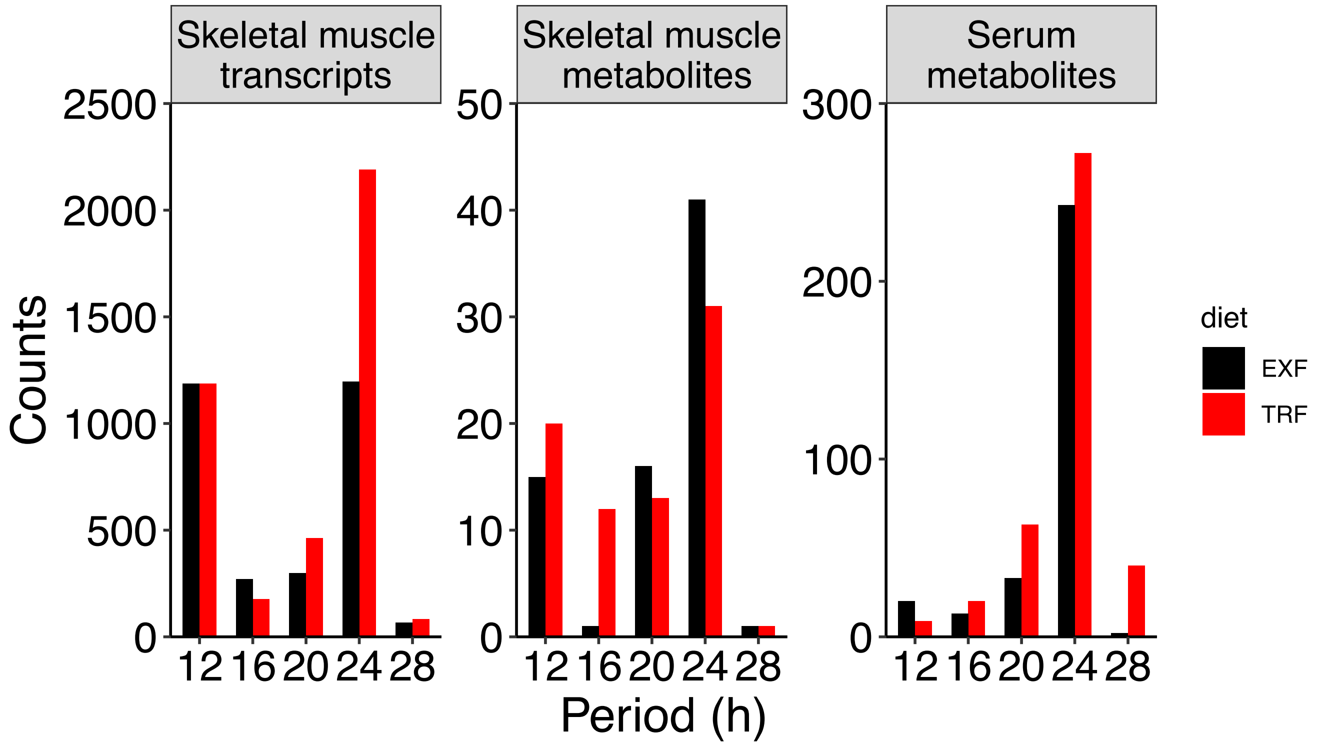

Supplementary Figure 2. Period distributions of rhythmic features. Periods of transcripts in skeletal muscle (left panel), metabolites in skeletal muscle (middle panel), and metabolites in serum (right panel). Black indicates extended feeding (EXF), and time-restricted feeding (TRF). n = 11, source data are provided as a Source Data file.

Supplementary Figure 3

a

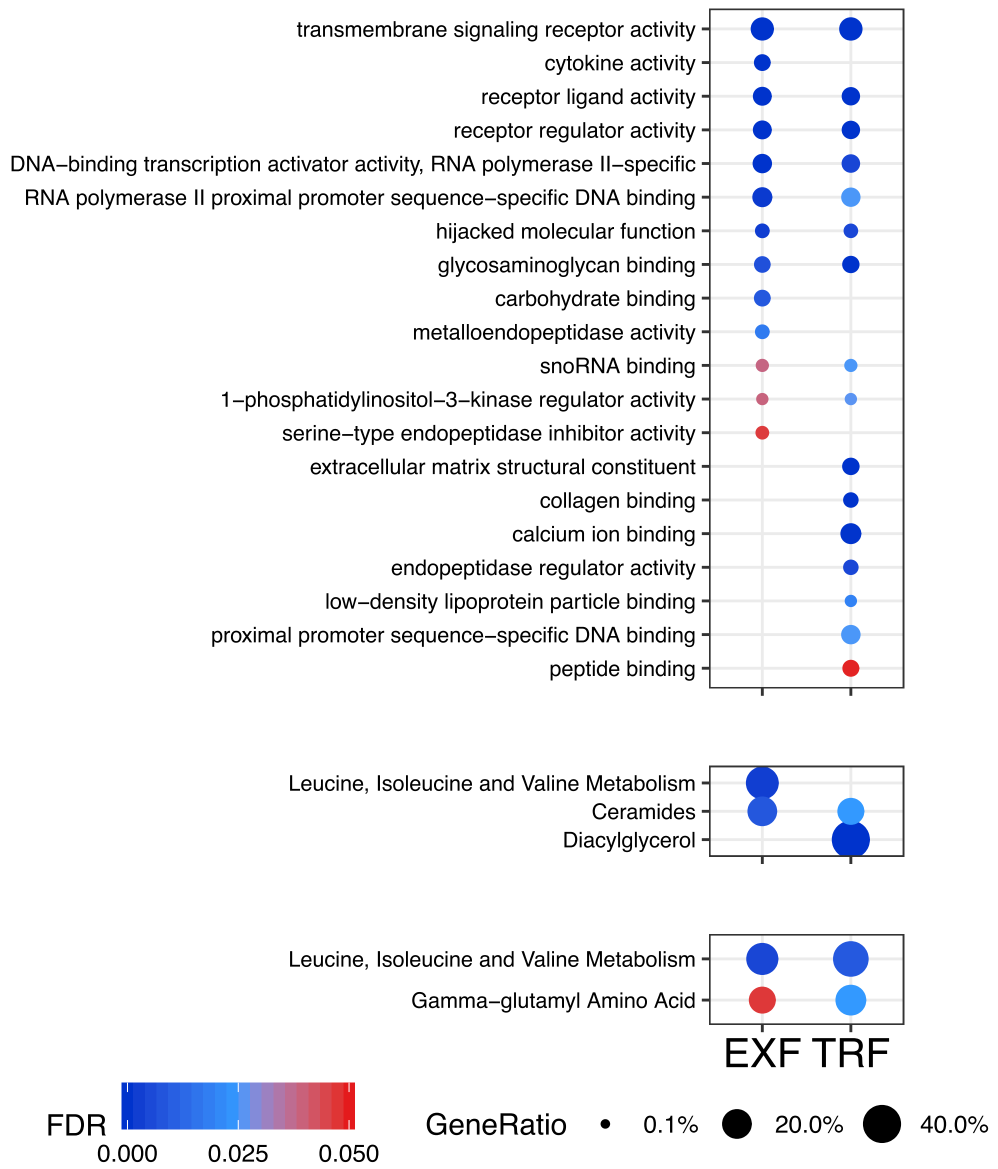

b

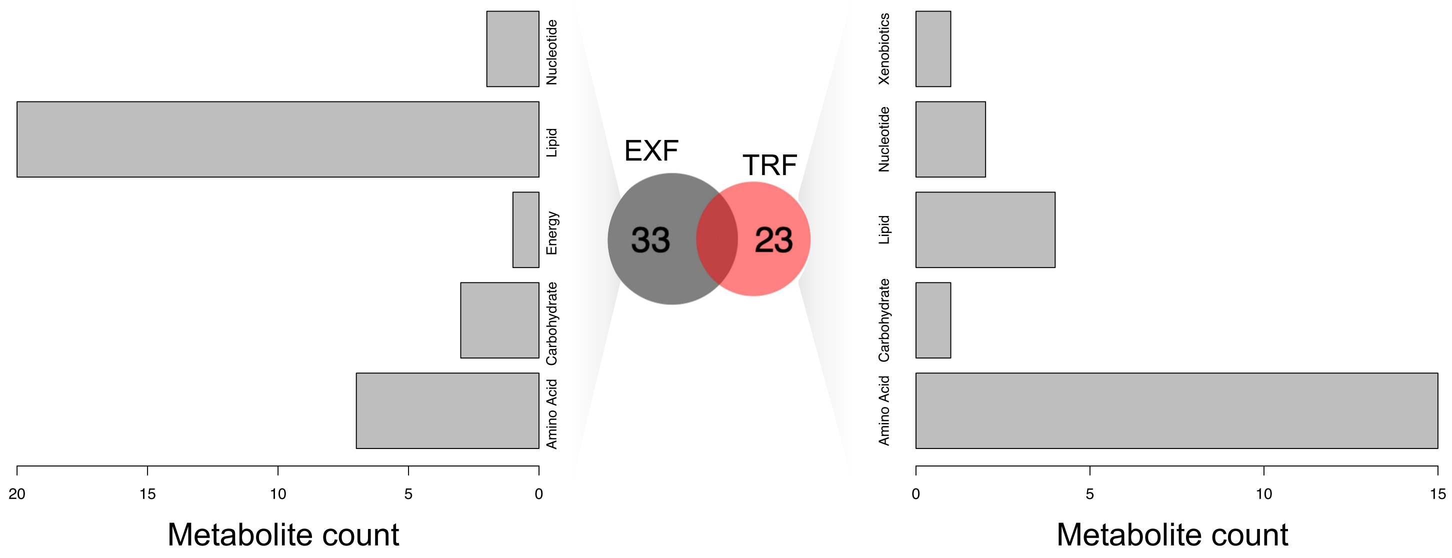

Supplementary Figure 3. Enrichments of ultradian features. Overrepresentation analysis of molecular function gene ontology in ultradian (12h periodic) skeletal muscle periodic transcripts (top panel), and Metabolon sub-pathway definition of skeletal muscle metabolites (middle panel), and serum metabolites (bottom panel) after unrestricted feeding (EXF) and time-restricted feeding (TRF) (a). Color indicates FDR adjusted p value, circle size indicates the proportion of periodic genes in the ontology. Proportion of super-pathway definition in the unique skeletal muscle metabolites for EXF and TRF (b). n = 11 participants, source data are provided as a Source Data file.

Supplementary Figure 4

<u>Schematic bioinformatical approach</u>

Transcriptomics: preprocessing of transcriptomic data using STAR aligner and subRead. EdgeR to filter our transcripts expressed less than 10 in either group, and generate counts per million.

Metabolomics: Imputation of missing values for metabolomics.

All data: Power transformation of data

↓

Rain to identify features with period of 12-28h

↓

Retain features with periods = 24 or 12h

↓

CircaCompare to analyze differences in amplitude, MESOR, and acrophase only in features with 24 h periods in both TRF and EXF.

Supplementary Figure 4. Schematic of bioinformatics pipeline.
