# Supplementary material for: Time-restricted feeding alters lipid and amino acid metabolite rhythmicity without perturbing clock gene expression
Source: Nat Commun. 2020 Sep 16;11:4643. doi: 10.1038/s41467-020-18412-w (PMC7495469; doi:10.1038/s41467-020-18412-w)
Supplement: Supplementary file 2 — Description of Additional SupplementaryFiles [file 41467_2020_18412_MOESM2_ESM.pdf]

Description of Additional Supplementary Files

File Name: Supplementary Data 1

Description: Summary statistics of all rhythmic features. Data includes, RAIN derived p-values, FDR adjusted p-values, estimated period, MESOR, Amplitude, and acrophase

File Name: Supplementary Data 2

Description: Skeletal muscle and serum metabolite raw data.
